# A Nomogram Prediction Model and Validation of Hypoglycemia Risk in Patients With Decompensated Cirrhosis and Type 2 Diabetes

**DOI:** 10.1155/jdr/6812038

**Published:** 2026-04-16

**Authors:** Hui Liu, Yang Liu, Qiong Tian, Xiling Hu, Yide Li, Chunfei Wang

**Affiliations:** ^1^ Department of Nursing, The Third Affiliated Hospital of Sun Yat-sen University, Guangzhou, China, zssy.com.cn; ^2^ Endoscopy Center, The Seventh Affiliated Hospital, Sun Yat-sen University, Shenzhen, China, sysu.edu.cn; ^3^ Department of Endocrinology and Metabolism, The Third Affiliated Hospital of Sun Yat-sen University, Guangzhou, China, zssy.com.cn; ^4^ Department of Critical Care Medicine, The Seventh Affiliated Hospital, Sun Yat-sen University, Shenzhen, China, sysu.edu.cn

**Keywords:** liver cirrhosis, nomogram, predictive model, type 2 diabetes

## Abstract

**Purpose:**

This research sought to develop and evaluate a predictive nomogram for determining hypoglycemia likelihood in cases of concurrent decompensated cirrhosis and type 2 diabetes.

**Methods:**

This investigation included a cohort of 830 individuals with decompensated cirrhosis and type 2 diabetes in a retrospective design. A random 7:3 split was carried out to create model training and validation sets. Key predictors were selected using LASSO regression followed by multivariate logistic regression to construct the nomogram. Evaluation of the model encompassed both discrimination, quantified by the area under the receiver operating characteristic (ROC) curve (AUC), and calibration, assessed via calibration curves. The model’s generalizability was further confirmed using the held‐out validation set.

**Results:**

Among the 830 participants included in the analysis, hypoglycemia occurred in 118 cases, corresponding to an incidence rate of 14.22%. Eight variables were significantly associated with hypoglycemia: diabetic peripheral neuropathy, creatinine (Cr), indirect bilirubin (IB), triglycerides (TGs), use of antibiotics within 48 h of admission, duration of diabetes, insulin dosage, and body mass index (BMI). In the training set, the model’s discriminative ability was substantial, with an AUC of 0.736 (95% CI: 0.680–0.792). Notably, this performance was well‐maintained in the validation set (AUC = 0.709,95% CI: 0.614–0.803), with reliable calibration demonstrated through strong concordance between model forecasts and actual results.

**Conclusions:**

We have constructed a nomogram that incorporates eight predictors to estimate hypoglycemia risk in patients with decompensated cirrhosis and type 2 diabetes. It serves as a practical instrument for hypoglycemia risk assessment, providing valuable support for clinical decisions aimed at prevention and management strategies.

**Summary**

• This study revealed eight independent predictors connected with hypoglycemia in individuals with decompensated cirrhosis and type 2 diabetes.
• A nomogram was constructed through these factors to effectively stratify high‐risk patients, enabling targeted prevention and clinical management.
• Adjusting of three potentially modifiable variables, including use of antibiotics within 48 h of admission, insulin dosage, and body mass index (BMI), may help mitigate hypoglycemia incidence.

## 1. Introduction

Liver cirrhosis and type 2 diabetes have a high global prevalence, leading to approximately 1 million deaths annually and affecting over 400 million individuals worldwide, respectively [1, 2]. The two conditions frequently coexist, as factors like comorbidities, acute medical events, and medication use can significantly disrupt glucose metabolism. Additionally, the presence of diabetes elevates the risk of complications associated with cirrhosis [3, 4]. Prior studies have shown that their co‐occurrence increases hypoglycemia risk, particularly severe episodes, by about 2.7 times compared to diabetes alone [5, 6]. Hypoglycemia threatens the life, health, and quality of life of patients with cirrhosis and type 2 diabetes, ranging from mild symptoms such as sweating, palpitations, and insomnia to severe outcomes including multiorgan dysfunction, impaired consciousness, and even death [7–9]. Additionally, hypoglycemia exacerbated the economic burden on patients [10].

Patients with cirrhosis combined with type 2 diabetes are considered a high‐risk group for hypoglycemia. Studies indicate that several clinical parameters can contribute to this risk, including age, gender, body mass index (BMI), diabetes duration, renal dysfunction, disease stage, and insulin use [8, 11–13]. However, previous researches have mainly focused on hypoglycemia in diabetic patients without addressing the unique aspects of those with both cirrhosis and type 2 diabetes. The mechanism of hypoglycemia in cirrhosis fundamentally differs from that in diabetes alone. As the central organ in glucose metabolism, the liver exhibits severely impaired glycogen synthesis, storage, and gluconeogenic capacity in cirrhosis, leading to fragile systemic glucose homeostasis [14]. Liver cirrhosis can also affect the absorption, distribution, metabolism, elimination, and bioavailability of hypoglycemic drugs [15]. Furthermore, the portosystemic shunting of liver cirrhosis may cause hypoglycemia in patients [16]. Insulin resistance is a key metabolic factor influencing glucose homeostasis. It has been demonstrated that the METS‐IR, as a marker for insulin resistance, serves as a significant predictor for the development of type 2 diabetes in nonobese populations [17]. In patients with decompensated cirrhosis and type 2 diabetes, factors such as the liver disease itself, portosystemic shunting, and chronic inflammation may further exacerbate insulin resistance. This, synergizing with already compromised hepatic function, collectively increases the risk of abnormalities in glucose utilization and regulation, forming a distinct pathophysiological mechanism for hypoglycemia unique to this patient group.

This organ‐specific dysfunction reshapes the patient’s risk profile, suggesting that predictive biomarkers and risk determinants are context‐dependent. Similar to how a molecular determinant like CENPA exhibits distinct prognostic stratification across different cancer types, the clinical and metabolic drivers of hypoglycemia likely differ fundamentally between general diabetes and cirrhosis‐associated diabetes [18]. Consequently, prediction models developed from general diabetic cohorts may exhibit limited discriminative performance and clinical utility in cirrhotic populations, as they fail to capture the unique pathophysiological mechanisms underlying hepatic glucose dysregulation. This limitation also exists in risk prediction for other liver‐related conditions. For example, studies on predicting type 2 diabetes risk in patients with nonalcoholic fatty liver disease highlight the need for specific models. In these cases, directly applying generic tools is insufficient [19, 20]. This supports our view that risk prediction in diabetic patients with underlying liver diseases must consider the distinct impact of the liver condition.

Based on a prior analysis of clinical records from individuals diagnosed with both cirrhosis and type 2 diabetes, a heightened incidence of hypoglycemic events was observed among those with decompensated cirrhosis relative to their counterparts with compensated disease. Furthermore, hypoglycemia is well‐established as a clinically significant risk factor in intensive care populations [21]. Currently, there is a lack of a reliable approach to forecast hypoglycemic events in individuals suffering from decompensated cirrhosis accompanied by type 2 diabetes.

To address this gap, our study sought to develop a prediction tool for assessing hypoglycemia risk in this patient group. We created a nomogram designed to be user‐friendly, enabling clinicians to quickly and conveniently evaluate the probability of hypoglycemia in their patients.

## 2. Materials and Methods

### 2.1. Ethical approval

The research adhered to the ethical guidelines established in the Declaration of Helsinki(1975) and was approved by the Institutional Review Boards of The Third Affiliated Hospital (RG‐2023‐115‐01) and The Seventh Affiliated Hospital (KY‐2024‐404‐01) of Sun Yat‐sen University. Informed consent was waived for this retrospective analysis of anonymized patient data.

### 2.2. Participants

This study initially enrolled 830 patients diagnosed with both decompensated cirrhosis and type 2 diabetes from The Third and Seventh Affiliated Hospitals of Sun Yat‐sen University during 2018–2023. Individuals with the following conditions were included: (i) adult patients (≥18 years); (ii) meeting Chinese guideline criteria for decompensated cirrhosis [22]; (iii) fulfilling World Health Organization 1999 diagnostic standards for type 2 diabetes; (iv) having diabetes diagnosed concurrently with or after the cirrhosis diagnosis. Conversely, exclusion criteria were: (i) Pregnancy; (ii) surgical patients; (iii) those with a severe lack of clinical data.

### 2.3. Study Design

This retrospective investigation began with a preliminary identification of potential predictors for hypoglycemia through a literature review and expert consultation, which lead to the development of a dedicated data collection form. Patient information was subsequently extracted from both electronic and paper‐based medical records.

Data extraction from medical records included:

Demographics/history: sex, age, BMI, family history.
Medications: use of dipeptidyl peptidase‐IV (DPP‐4), sodium‐dependent glucose transporters‐2 (SGLT‐2) inhibitors, and so forth.
Laboratory parameters: alkaline phosphatase (ALP), aspartate aminotransferase (AST), alanine aminotransferase (ALT), γ‐glutamyl transpeptidase (γ‐GT), albumin (ALB), globulin (GLB), total protein (TP), prealbumin (PA), creatinine (Cr), total bilirubin (TB), direct bilirubin (DB), indirect bilirubin (IB), total cholesterol (TC), triglycerides (TGs), low‐density lipoprotein cholesterol (LDL‐C), high‐density lipoprotein cholesterol (HDL‐C), white blood cell (WBC) count, platelet (PLT) count, hemoglobin (Hb), hematocrit (Hct), prothrombin activity (PTA), activated partial thromboplastin time (APTT), prothrombin time (PT), thrombin time (TT), fibrinogen (FIB), and so forth.

Hypoglycemia was measured by venous plasma glucose value or capillary blood glucose monitoring value <3.9 mmol/L [23].

### 2.4. Statistical Analysis

Sample size estimation was performed according to the criteria proposed by Riley et al. [24]. Based on an outcome prevalence of 0.13, 10 candidate predictor parameters, and a conservative Cox–Snell *R* ^2^ of 0.2, the minimum sample size required for model development was calculated to be 398.

All analyses were carried out with R software, version 4.2.0. Continuous measures following normal distributions were described using mean ± standard deviation (SD) and subjected to *t*‐test analysis. Nonnormally distributed data are expressed as median with interquartile spans (*M* (Q25, Q75)) and examined via the Mann–Whitney *U* testing, represented numerically with proportional rates (*N* (%)), and group differences investigated using either chi‐square or Fisher’s exact tests, depending on application conditions. Variables with ≥30% missingness were excluded; others were imputed via the random forest algorithm. Postimputation assessment verified that data distributions remained consistent (Table S1 and Figure S1). Participants were subsequently randomized into training (70%) and validation (30%) sets. Predictors were selected using LASSO with tenfold cross‐validation, choosing lambda.min to maximize predictive performance. As LASSO addresses multicollinearity by design, no separate collinearity testing was performed. Variables retained by LASSO were entered into the multivariable logistic regression model. The model’s predictive accuracy was examined through two key aspects: discrimination capacity, measured by the area under the receiver operating characteristic (ROC) curve (AUC), and goodness‐of‐fit, evaluated using calibration curves and Hosmer–Lemeshow test. It should be noted that the latter has recognized limitations in large samples or under specific circumstances. Internal validity was assessed using a bootstrap resampling procedure with 1000 iterations to calculate the optimism‐corrected AUC and quantify potential overfitting. Statistical significance was defined at the 0.05 threshold.

## 3. Results

### 3.1. Patient Characteristics

As presented in Table 1, the final cohort comprised 830 eligible patients. During their hospitalization, 118 individuals (14.22%) experienced hypoglycemia, while 712 did not. Significant intergroup differences (*p* < 0.05) were observed for multiple variables: diabetic kidney disease, diabetic peripheral neuropathy, and diabetic vasculopathy prevalence; ALP, PA, Cr, and TG; along with the use of antibiotics within 48 h of admission, insulin dosage, diabetes duration, and BMI. The complete baseline data for all collected variables are provided in Table S2.

**Table 1:** Baseline characteristics in the hypoglycemia and nonhypoglycemia groups (key variables only).

Variables	All	Hypoglycemia		*p*
		No (*n* = 712) *n* (%)/*M*(Q25, Q75)	Yes (*n* = 118) *n* (%)/*M*(Q25, Q75)	
Age (year)	59 (52, 67)	59 (52, 67)	60 (53, 66)	0.9
Sex				0.6
Male	643 (77%)	554 (78%)	89 (75%)	—
Female	187 (23%)	158 (22%)	29 (25%)	—
Family history of diabetes				0.7
No	773 (93%)	664 (93%)	109 (92%)	—
Yes	57 (6.9%)	48 (6.7%)	9 (7.6%)	—
Hydrothorax				0.8
No	711 (86%)	611 (86%)	100 (85%)	—
Yes	119 (14%)	101 (14%)	18 (15%)	—
Ascites				0.4
No	379 (46%)	329 (46%)	50 (42%)	—
Yes	451 (54%)	383 (54%)	68 (58%)	—
Diabetic kidney disease				<0.001
No	749 (90%)	653 (92%)	96 (81%)	—
Yes	81 (9.8%)	59 (8.3%)	22 (19%)	—
Diabetic peripheral neuropathy				<0.001
No	763 (92%)	664 (93%)	99 (84%)	—
Yes	67 (8.1%)	48 (6.7%)	19 (16%)	—
Diabetic peripheral vasculopathy				0.007
No	741 (89%)	644 (90%)	97 (82%)	—
Yes	89 (11%)	68 (9.6%)	21 (18%)	—
ALP (U/L)	121 (89, 177)	119 (88, 171)	133 (99, 195)	0.007
PA (mg/L)	86 (52, 131)	86 (54, 132)	70 (41, 127)	0.027
Cr (μmol/L)	68 (54, 92)	67 (54, 90)	75 (57, 110)	0.006
IB (μmol/L)	13 (7, 27)	13 (7, 27)	12 (6, 30)	0.5
TG (mmol/L)	1.13 (0.84, 1.63)	1.15 (0.86, 1.65)	1.02 (0.77, 1.36)	0.006
Antibiotics within 48 h				0.022
No	699 (84%)	608 (85%)	91 (77%)	—
Yes	131 (16%)	104 (15%)	27 (23%)	—
Insulin dosage	18 (0, 32)	16 (0, 32)	30 (17, 43)	<0.001
Duration of diabetes (year)	18 (1, 33)	18 (1, 30)	21 (14, 42)	<0.001
BMI (kg/m^2^)	23.4 (21.3, 25.9)	23.8 (21.4, 26.0)	22.3 (20.5, 24.2)	<0.001

### 3.2. Identification of Predictive Factors

**Figure 1:**
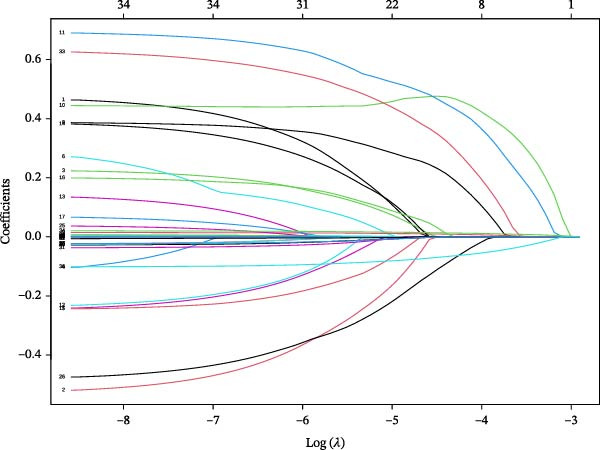
LASSO coefficient distribution for all risk factors.

**Figure 2:**
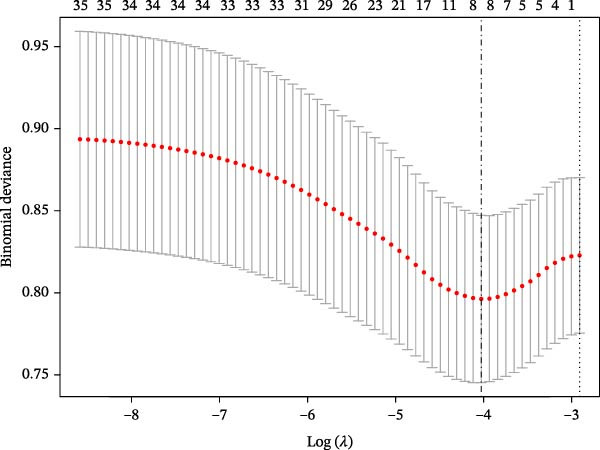
Optimal predictor (lambda) selection in the LASSO model. The left and right dashed vertical lines correspond to the values of lambda at the minimum error (lambda.min) and at standard error (lambda.1−SE), respectively.

**Figure 3:**
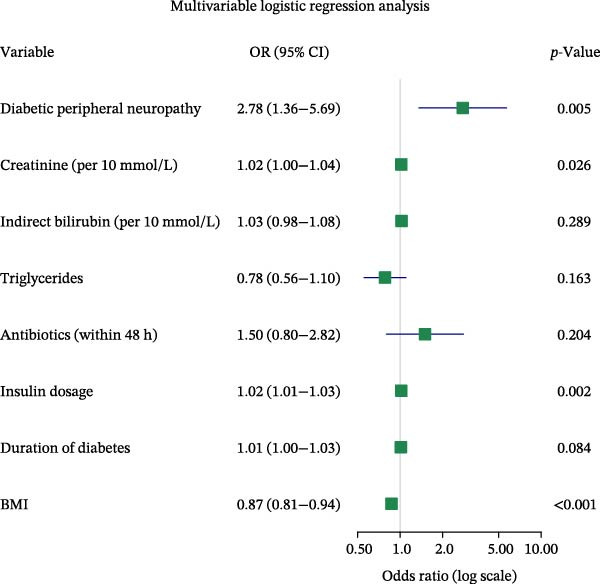
Multivariate logistic regression analysis of the eight predictors in the hypoglycemia prediction model.

Univariate analysis showed no significant association between IB and the outcome (*p* > 0.05). Eight nonzero variables were selected by LASSO regression, including diabetic peripheral neuropathy, Cr, IB, TGs, use of antibiotics within 48 h of admission, insulin dosage, duration of diabetes, and BMI (Figures 1 and 2). A final model was developed through multivariate logistic regression, incorporating the eight variables. Among these, TGs and BMI were identified as protective factors, while the others were recognized as risk factors. The complete multivariate analysis is provided in Figure 3.

### 3.3. Development of the Prediction Model

**Figure 4:**
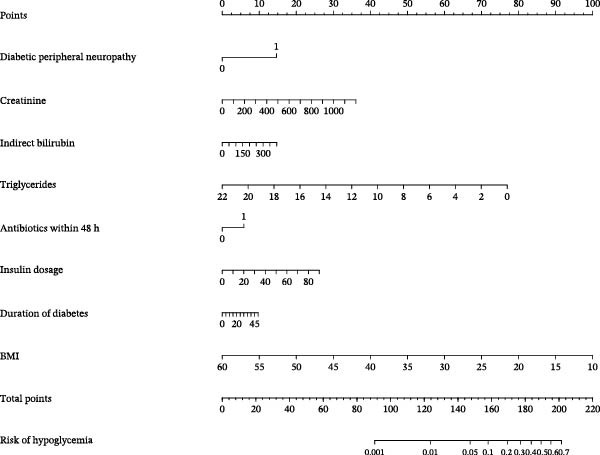
Nomogram and scoring method for hypoglycemia in patients with decompensated cirrhosis and type 2 diabetes.

We developed a nomogram to predict the probability of hypoglycemia by incorporating eight significant clinical predictors identified previously (Figure 4). Detailed statistical results of the final multivariable model, including regression coefficients and standard errors, are provided in Table S3. This tool visually depicts the relative weight of each factor. To use it, a score is assigned at the top scale based on the individual’s value for each predictor. The summation of all partial scores generates a total points value. This score is projected downward to the bottom scale, where the corresponding predicted probability of hypoglycemia can be read.

### 3.4. Predictive Model Validation

#### 3.4.1. Discrimination

**Figure 5:**
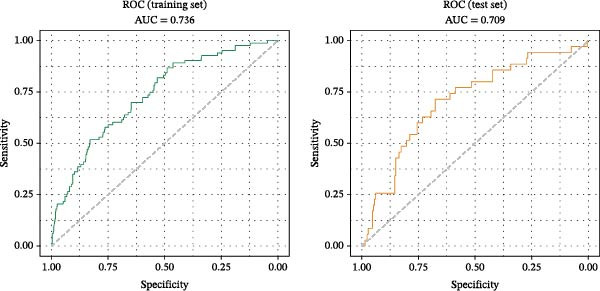
The ROC curve and corresponding AUC in the training set and test set.

Evaluation of the nomogram’s discrimination demonstrated an AUC value of 0.736 (95% CI: 0.680–0.792) in the training set. The validation set yielded a comparable AUC of 0.709 (95% CI: 0.614–0.803; Figure 5), demonstrating the model’s moderate discriminative power.

#### 3.4.2. Calibration of the Predictive Model

**Figure 6:**
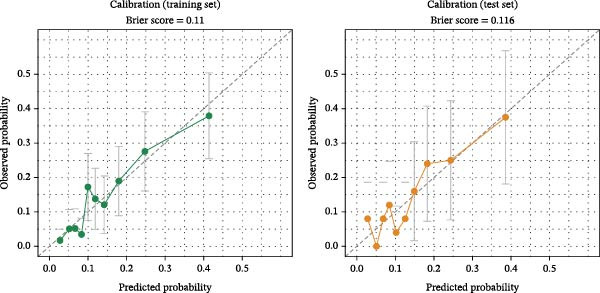
Calibration curves for the hypoglycemia predictive model in the training set and test set.

Furthermore, calibration analysis revealed no significant deviation between predictions and observations, as evidenced by the calibration curves (Figures 6). Hosmer–Lemeshow test showed no significant deviation (*p*‐values = 0.74 and 0.57), collectively confirming acceptable agreement between model forecasts and empirical data. Internal validation via 1000 bootstrap resamples confirmed model robustness. The analysis revealed a marginal optimism of 0.024, resulting in a corrected AUC of 0.712 compared to the original value of 0.736.

## 4. Discussion

In patients with decompensated cirrhosis concomitant with type 2 diabetes, the occurrence of hypoglycemia arises from the combined effects of multiple pathophysiological mechanisms. The predictive nomogram developed in this study incorporates eight variables, collectively depicting different dimensions of this systemic risk: Cr and IB indicate hepatic and renal dysfunction and decreased hepatocellular functional reserve; BMI and duration of diabetes may reflect insufficient energy reserves due to long‐term metabolic disturbances and malnutrition; antibiotic use signals interference with metabolic homeostasis from infection or systemic inflammation; insulin dose represents a direct iatrogenic risk factor; diabetic peripheral neuropathy may reduce the perception and regulatory capacity for hypoglycemia by impairing autonomic nerve function. By integrating indicators that reflect organ function, metabolic status, inflammation level, and treatment intensity, this model aims to comprehensively assess overall metabolic vulnerability in patients. The model achieved AUCs of 0.736 and 0.709 in the training and validation sets, respectively, indicating moderate and stable discriminative ability, with agreement between predicted probabilities and actual event rates. This suggests that the tool offers a multidimensional, integrated risk assessment framework for identifying high‐risk patients clinically.

Further, it is worth noting that cirrhosis is not only a metabolic disease but also closely related to systemic inflammation and immune dysregulation. In the related field, even in transplantation medicine, integrative models outperform single biomarkers. Research shows that the mechanisms by which traditional Chinese medicine supports liver transplantation recovery are related to its regulation of systemic inflammation and enhancement of overall metabolic resilience. These systemic factors have a far greater impact on clinical outcomes than any single laboratory parameter [25]. This supports the core idea of this study: the risk of hypoglycemia is the result of the combined effects of liver and kidney function, energy reserves, neural regulation, inflammatory status, and therapeutic intervention.

The eight predictors screened out by this model are not isolated risk markers, but rather are interrelated and collectively form a risk network that reflects systemic metabolic decompensation.

Our results identified diabetic peripheral neuropathy as an independent determinant for hypoglycemia development, consistent with previous research [26]. This diabetic microvascular complication exhibits 67.6% prevalence in type 2 diabetes populations, demonstrating a gradual increase proportional to aging and disease duration [27, 28]. In decompensated cirrhosis, factors such as malnutrition and vitamin deficiencies, particularly vitamin B due to impaired hepatic synthesis, may worsen diabetic peripheral neuropathy by compromising nerve repair [29]. Diabetic peripheral neuropathy is characterized by distal sensory axon apoptosis and disrupted intracellular transport [30, 31], typically presenting as symmetric stocking‐glove neuropathy with reduced protein synthesis in dorsal root ganglia [32, 33]. This impairment affects peripheral and autonomic nerves, diminishing the perception of and response to hypoglycemia [34, 35], thereby increasing the risk of severe hypoglycemia. Conversely, hypoglycemia itself can induce distal axonal damage, and even mild episodes may contribute to neuropathy [36], suggesting that early peripheral neuropathy may also indicate prior hypoglycemic occurrences.

In this research, higher Cr levels were linked to hypoglycemia risk. As a direct marker of renal function, Cr reflects kidney impairment [37]. It is important to emphasize that renal dysfunction is extremely common in patients with decompensated cirrhosis. Under normal conditions, both the liver and kidneys participate in hypoglycemic counterregulation via gluconeogenesis [38, 39]. However, in decompensated cirrhosis, impaired hepatic function limits the liver’s compensatory role, increasing dependence on renal glucose release during hypoglycemia [40]. Therefore, the predictive weight of Cr in this model likely reflects more the severe disruption of the “hepatorenal interaction,” a core pathophysiological axis. Additionally, renal insufficiency may lead to hypoglycemia through reduced clearance of hypoglycemic agents (including insulin), anorexia, malnutrition, and treatment‐related side effects [41, 42]. When renal function is concurrently impaired—as indicated by elevated Cr—the ability to compensate through gluconeogenesis is compromised [43]. Against the backdrop of hepatic gluconeogenesis failure caused by cirrhosis, renal dysfunction suggests that the final compensatory glucose‐producing pathway is also impaired, potentially accompanied by the risk of drug accumulation. Thus, Cr should be understood more as a key and easily available marker for identifying patients at high risk of “systemic homeostatic decompensation.”

Notably, IB, as one of the predictors in the final model, was not significant in univariate analysis. This suggests that the predictive value of IB for hypoglycemia risk may mainly emerge in the context of multivariable synergistic effects. IB was a risk factor of hypoglycemia in our study, which previous studies have not discovered. This may be linked to insulin sensitivity. Previous research has shown that serum IB may act as a protective factor that improves insulin sensitivity [44]. Enhanced insulin sensitivity may facilitate accelerated glucose clearance from the bloodstream, consequently increasing vulnerability to hypoglycemic events. Additionally, in patients with decompensated cirrhosis, IB levels may reflect bile excretion function and may also be associated with a general decline in the liver cells’ ability to process hormones or substrates related to glucose metabolism. Therefore, the retention of IB in the model may indicate a state associated with overall metabolic dysfunction of hepatocytes.

Furthermore, TGs are important energy reserves of the body, which can be decomposed into fatty acids and glycerol in a hypoglycemic state, and participate in gluconeogenesis and energy supply, respectively [45]. Their role must be considered when assessing hypoglycemia risk in liver disease. Patients with cirrhosis often suffer from lipid metabolism disorders, including low TGs, low cholesterol, and hypoalbuminemia [46], which together are part of liver‐related malnutrition or a “catabolic metabolic state.” In this context, low TG levels may signal two things. First, severe energy reserve depletion leaves too few lipid substrates to support gluconeogenesis from glycerol and to provide alternative energy from fatty acids during stress or fasting. Second, low TG levels may indirectly mark a broadly impaired hepatic synthetic function. Thus, TGs are not an isolated “hypoglycemia factor.” Instead, they are part of a high‐risk metabolic state defined by systemic energy failure and reduced hepatic synthesis.

This model included variables such as IB and TGs. Compared to predictors such as insulin dose, the specific association of these variables with hypoglycemia has been shown to have limited and inconsistent evidence in previous studies. In the context of decompensated liver cirrhosis with type 2 diabetes, IB and TG may not be direct causal factors, but rather reflect a broader state of severe hepatocellular dysfunction and systemic energy metabolism depletion. Their inclusion in the model suggests that they may serve as markers for identifying patients in a vulnerable and multisystem metabolic decompensated state, rather than establishing a direct causal link to hypoglycemia.

Our study identified antibiotic use within 48 h of admission as a significant predictor for hypoglycemia in patients, reflecting a clinical presumption of community‐acquired infection. Infection is strongly associated with hypoglycemia, which is a frequent manifestation of septicemia in cirrhotic patients and an initial autonomous predictor of bloodstream infections and inpatient fatality [47, 48]. Although infection more commonly leads to hyperglycemia, it can also cause hypoglycemia in severe systemic responses such as sepsis [49, 50]. The mechanisms underlying infection‐related hypoglycemia are not fully elucidated but may involve several pathways, including a decline in cortisol and adrenaline levels impairing glycemic maintenance, altered glucose metabolism due to failed hepatic gluconeogenesis and increased peripheral glucose consumption [50–52], and inflammatory‐mediated disruption of hepatic CRTC2 signaling [53].

Insulin dosage was also identified as a contributing factor to hypoglycemia development in our cohort, with a direct association observed between dosage increments and hypoglycemia probability. This observation is consistent with the previous report, which indicated that higher hypoglycemia rates in patients receiving 1.1 U/kg insulin relative to those administered 0.6 U/kg [54]. Moreover, the frequency and timing of insulin administration are also known to influence the risk of hypoglycemia [55, 56].

Several studies have shown that the duration of diabetes correlates with the risk of hypoglycemia, with an extended disease course predicting heightened vulnerability to hypoglycemic events [57, 58]. One possible reason is that as diabetes progresses, the body’s counter‐regulatory response to hypoglycemia gradually deteriorates [59]. A fundamental contributing mechanism involves the progressive impairment of glucagon’s counter‐regulatory response during insulin‐mediated hypoglycemic episodes. The loss of the protective hormonal response can also lead to the symptoms of hypoglycemia becoming less pronounced overtime [60].

It has been confirmed that BMI is significantly correlated with blood glucose levels [61, 62]. Patients who experience hypoglycemic episodes had lower BMI than those who do not experience hypoglycemic episodes [63]. A study found that among 81 patients experiencing hypoglycemia, the majority (49.40%) had a BMI ranging from 18 to 22.9 kg/m^2^, which is considered average weight according to Asian standards [10]. Among recently diagnosed diabetic individuals, a lower BMI correlates with increased blood glucose variability, making them more prone to hypoglycemia [64]. The severity of hypoglycemic episodes also appears to be influenced by BMI. The study documented significantly more pronounced plasma glucose reduction in low‐BMI participants during iatrogenic hypoglycemic challenges compared to high‐BMI subjects [65].

Compared to existing hypoglycemia prediction models for general type 2 diabetes patients [66, 67], Yang et al.’s [66] machine learning model in hospitalized type 2 diabetes patients (external validation AUC 0.82) [66], and Chen et al.’s [67] logistic regression–based model for community‐dwelling elderly type 2 diabetes patients (internal validation AUC 0.8), both demonstrated good predictive performance. Another study using the XGBoost algorithm in hospitalized type 2 diabetes patients achieved even higher discriminative ability (internal validation AUC 0.910) [68]. Although these models performed well in their respective cohorts, they were all developed based on general or elderly type 2 diabetes populations and did not account for the unique pathophysiological context of liver cirrhosis.

The nomogram constructed in this study showed moderate discriminative ability in internal validation, and we fully acknowledge this statistical performance gap compared to the aforementioned models. However, when evaluating its clinical value, it should be considered within the specific context of “decompensated cirrhosis combined with type 2 diabetes,” for which dedicated risk prediction tools are currently lacking. The key predictors and their weighting in existing high‐performance models may not adequately capture the distinct mechanisms of glucose metabolism disorders in the setting of cirrhosis.

Therefore, the core value of our model lies in its disease specificity and clinical integration. By systematically incorporating and quantifying eight routinely available clinical variables, it provides—for the first time—a standardized and structured risk assessment framework for this high‐risk population. This helps overcome the current limitations in clinical practice, which rely heavily on physician experience and result in inconsistent assessments.

In real‐world clinical settings, the primary role of a prediction model with moderate discriminative ability is not to provide an absolute individual diagnosis, but to enable risk‐stratification and targeted allocation of preventive resources. By using this nomogram to identify patients with higher risk scores, clinical teams can implement intensified interventions, such as increasing the frequency of bedside blood glucose monitoring, optimizing nutritional support, carefully adjusting insulin dosages, or prioritizing hypoglycemia‐lowering glucose‐lowering medications. For patients with lower scores, unnecessary excessive monitoring and interventions can be avoided. This risk‐based differential management strategy has the potential to reduce the incidence of severe hypoglycemic events at the population level while improving the efficiency of healthcare resource utilization. Thus, the utility of this tool lies in providing a quantitative basis for structured clinical decision making.

However, this study has certain limitations that warrant acknowledgment. First, as the data were derived from two affiliated hospitals of the same university in China and the model was primarily evaluated on internal datasets, the external validity of the results may be limited. Differences in regions, healthcare systems, and population characteristics (such as disease composition and treatment protocols) may affect the model’s generalizability. Second, this study used a retrospective design, which involves potential selection and information bias: patients with severe conditions or frequent hospitalizations are more likely to be included, while the characteristics of mild cases remain unknown; variables that were not routinely measured at admission may be missing; information on treatments such as insulin dosage and antibiotic use relies on medical records and may be subject to measurement errors due to incomplete documentation. These factors weaken the ability to infer causality, and unmeasured confounding factors cannot be ruled out; the observed associations remain at the statistical level and require verification for causality and generalizability in prospective multicenter studies. Additionally, the biological plausibility of certain predictors in the model (such as IB and TGs) in relation to hypoglycemia risk has not been fully established, and their associations require further validation in future studies. Finally, since the study population consisted solely of inpatients, with no data collected from nonhospitalized individuals such as community‐based populations, the broader applicability of the results remains to be further investigated.

Therefore, we plan to collaborate with domestic and international centers to conduct multicentre and prospective cohort studies that will assess the model’s robustness and applicability across different clinical scenarios. Additionally, we will investigate its utility in primary care institutions and within various national health systems to confirm its real‐world applicability. After completing the above external validation, we plan to develop a web‐based interactive calculator for clinicians to quickly use through a browser in the clinic or ward. At the same time, we will also explore the possibility of simple integration with hospital electronic medical record systems to enable partial automatic data entry, further enhancing efficiency.

## 5. Conclusions

We constructed a predictive nomogram specifically designed to assess hypoglycemia risk in individuals diagnosed with both decompensated hepatic cirrhosis and type 2 diabetes. The nomogram incorporates eight preoperative predictor variables and demonstrated potential for clinical application in internal validation. However, the data for this study come from a single academic medical center system, and the external validity of the model still needs to be further verified in different populations and medical settings. In the future, multicenter and prospective studies are needed to externally validate and refine the model, thereby enhancing its generalizability and clinical applicability. The nomogram is helpful in predicting hypoglycemia and can aid clinicians in determining the necessity for preemptive measures through integrated evaluation of prediction outcomes and patient‐specific factors.

## Author Contributions

**Hui Liu:** methodology, data curation, writing – original draft. **Yang Liu:** formal analysis, data curation, writing – original draft. **Qiong Tian:** investigation, methodology, writing – original draft. **Xiling Hu:** supervision, project administration. **Yide Li:** formal analysis, conceptualization, writing – review and editing. **Chunfei Wang:** funding acquisition, supervision, conceptualization, writing – review and editing.

## Funding

This study was funded by the Guangdong Provincial Medical Science and Technology Research Fund (Grant B2025281) and supported by the Sanming Project of Medicine in Shenzhen (Grant SZSM202311017).

## Disclosure

All authors contributed to the article and approved the submitted version.

## Conflicts of Interest

The authors declare no conflicts of interest.

## Supporting Information

Additional supporting information can be found online in the Supporting Information section.

## Supporting information


**Supporting Information** Table S1. Sensitivity analysis comparing the descriptive statistics (median and IQR) of continuous variables before and after random forest imputation. Table S2. Baseline characteristics in the hypoglycemia and nonhypoglycemia groups. Table S3. Multivariable logistic regression analysis of risk factors associated with hypoglycemia. Figure S1. Density plot comparisons of variables with missing values before and after imputation.

## Data Availability

The data presented in this study are available upon request from the corresponding authors.
