# Supplementary material for: A Nomogram Prediction Model and Validation of Hypoglycemia Risk in Patients With Decompensated Cirrhosis and Type 2 Diabetes
Source: J Diabetes Res. 2026 Apr 16;2026:6812038. doi: 10.1155/jdr/6812038 (PMC13087448; doi:10.1155/jdr/6812038)

**Supplementary Table S1.** Sensitivity analysis comparing the descriptive statistics (Median and IQR) of continuous variables before and after Random Forest imputation.

|  | **Original Data (with NAs)** | | **Imputed Data (missForest)** | |
| --- | --- | --- | --- | --- |
| **Characteristic** | **N** | **N = 830**^1^ | **N** | **N = 830**^1^ |
| **Age(year)** | 830 |  | 830 |  |
| Median (Q1, Q3) |  | 59 (52, 67) |  | 59 (52, 67) |
| Missing |  | 0 |  |  |
| **Sex** | 830 |  | 830 |  |
| 1 |  | 643 (77%) |  | 643 (77%) |
| 2 |  | 187 (23%) |  | 187 (23%) |
| Missing |  | 0 |  |  |
| **Family history of diabetes** | 830 |  | 830 |  |
| 0 |  | 773 (93%) |  | 773 (93%) |
| 1 |  | 57 (6.9%) |  | 57 (6.9%) |
| Missing |  | 0 |  |  |
| **Hypertension** | 830 |  | 830 |  |
| 0 |  | 547 (66%) |  | 547 (66%) |
| 1 |  | 283 (34%) |  | 283 (34%) |
| Missing |  | 0 |  |  |
| **Stroke** | 830 |  | 830 |  |
| 0 |  | 793 (96%) |  | 793 (96%) |
| 1 |  | 37 (4.5%) |  | 37 (4.5%) |
| Missing |  | 0 |  |  |
| **Cancer** | 830 |  | 830 |  |
| 0 |  | 542 (65%) |  | 542 (65%) |
| 1 |  | 288 (35%) |  | 288 (35%) |
| Missing |  | 0 |  |  |
| **Heart failure** | 830 |  | 830 |  |
| 0 |  | 789 (95%) |  | 789 (95%) |
| 1 |  | 41 (4.9%) |  | 41 (4.9%) |
| Missing |  | 0 |  |  |
| **Liver cancer** | 830 |  | 830 |  |
| 0 |  | 600 (72%) |  | 600 (72%) |
| 1 |  | 230 (28%) |  | 230 (28%) |
| Missing |  | 0 |  |  |
| **Hydrothorax** | 830 |  | 830 |  |
| 0 |  | 711 (86%) |  | 711 (86%) |
| 1 |  | 119 (14%) |  | 119 (14%) |
| Missing |  | 0 |  |  |
| **Ascites** | 830 |  | 830 |  |
| 0 |  | 379 (46%) |  | 379 (46%) |
| 1 |  | 451 (54%) |  | 451 (54%) |
| Missing |  | 0 |  |  |
| **Diabetic kidney disease** | 830 |  | 830 |  |
| 0 |  | 749 (90%) |  | 749 (90%) |
| 1 |  | 81 (9.8%) |  | 81 (9.8%) |
| Missing |  | 0 |  |  |
| **Diabetic peripheral neuropathy** | 830 |  | 830 |  |
| 0 |  | 763 (92%) |  | 763 (92%) |
| 1 |  | 67 (8.1%) |  | 67 (8.1%) |
| Missing |  | 0 |  |  |
| **Diabetic peripheral vasculopathy** | 830 |  | 830 |  |
| 0 |  | 741 (89%) |  | 741 (89%) |
| 1 |  | 89 (11%) |  | 89 (11%) |
| Missing |  | 0 |  |  |
| **Sulfonylurea** | 830 |  | 830 |  |
| 0 |  | 775 (93%) |  | 775 (93%) |
| 1 |  | 55 (6.6%) |  | 55 (6.6%) |
| Missing |  | 0 |  |  |
| **Metformin** | 830 |  | 830 |  |
| 0 |  | 706 (85%) |  | 706 (85%) |
| 1 |  | 124 (15%) |  | 124 (15%) |
| Missing |  | 0 |  |  |
| **Glycosidase inhibitors** | 830 |  | 830 |  |
| 0 |  | 714 (86%) |  | 714 (86%) |
| 1 |  | 116 (14%) |  | 116 (14%) |
| Missing |  | 0 |  |  |
| **SGLT-2** | 830 |  | 830 |  |
| 0 |  | 778 (94%) |  | 778 (94%) |
| 1 |  | 52 (6.3%) |  | 52 (6.3%) |
| Missing |  | 0 |  |  |
| **DPP-4** | 830 |  | 830 |  |
| 0 |  | 703 (85%) |  | 703 (85%) |
| 1 |  | 127 (15%) |  | 127 (15%) |
| Missing |  | 0 |  |  |
| **Beta blockers** | 830 |  | 830 |  |
| 0 |  | 689 (83%) |  | 689 (83%) |
| 1 |  | 141 (17%) |  | 141 (17%) |
| Missing |  | 0 |  |  |
| **Diuretic** | 830 |  | 830 |  |
| 0 |  | 374 (45%) |  | 374 (45%) |
| 1 |  | 456 (55%) |  | 456 (55%) |
| Missing |  | 0 |  |  |
| **Hormones** | 830 |  | 830 |  |
| 0 |  | 785 (95%) |  | 785 (95%) |
| 1 |  | 45 (5.4%) |  | 45 (5.4%) |
| Missing |  | 0 |  |  |
| **ALT(U/L)** | 828 |  | 830 |  |
| Median (Q1, Q3) |  | 33 (22, 65) |  | 33 (22, 65) |
| Missing |  | 2 |  |  |
| **AST(U/L)** | 828 |  | 830 |  |
| Median (Q1, Q3) |  | 47 (31, 88) |  | 47 (31, 87) |
| Missing |  | 2 |  |  |
| **ALP(U/L)** | 811 |  | 830 |  |
| Median (Q1, Q3) |  | 121 (89, 177) |  | 121 (90, 177) |
| Missing |  | 19 |  |  |
| **rGT(U/L)** | 814 |  | 830 |  |
| Median (Q1, Q3) |  | 85 (41, 173) |  | 88 (42, 173) |
| Missing |  | 16 |  |  |
| **TP(g/L)** | 827 |  | 830 |  |
| Median (Q1, Q3) |  | 67 (61, 73) |  | 67 (61, 73) |
| Missing |  | 3 |  |  |
| **ALB(g/L)** | 827 |  | 830 |  |
| Median (Q1, Q3) |  | 32.5 (28.6, 37.1) |  | 32.5 (28.7, 37.1) |
| Missing |  | 3 |  |  |
| **GLB(g/L)** | 826 |  | 830 |  |
| Median (Q1, Q3) |  | 34 (28, 39) |  | 34 (28, 39) |
| Missing |  | 4 |  |  |
| **PA(mg/L)** | 745 |  | 830 |  |
| Median (Q1, Q3) |  | 86 (52, 131) |  | 86 (54, 128) |
| Missing |  | 85 |  |  |
| **Cr(μmol/L)** | 826 |  | 830 |  |
| Median (Q1, Q3) |  | 68 (54, 92) |  | 68 (54, 92) |
| Missing |  | 4 |  |  |
| **TB(μmol/L)** | 823 |  | 830 |  |
| Median (Q1, Q3) |  | 23 (12, 59) |  | 23 (12, 59) |
| Missing |  | 7 |  |  |
| **DB(μmol/L)** | 823 |  | 830 |  |
| Median (Q1, Q3) |  | 9 (4, 28) |  | 9 (4, 28) |
| Missing |  | 7 |  |  |
| **IB(μmol/L)** | 817 |  | 830 |  |
| Median (Q1, Q3) |  | 13 (7, 27) |  | 13 (7, 27) |
| Missing |  | 13 |  |  |
| **TC(mmol/L)** | 748 |  | 830 |  |
| Median (Q1, Q3) |  | 3.95 (3.00, 4.95) |  | 3.97 (3.04, 4.90) |
| Missing |  | 82 |  |  |
| **TG(mmol/L)** | 747 |  | 830 |  |
| Median (Q1, Q3) |  | 1.13 (0.84, 1.63) |  | 1.18 (0.87, 1.63) |
| Missing |  | 83 |  |  |
| **LDL-c(mmol/L)** | 748 |  | 830 |  |
| Median (Q1, Q3) |  | 2.15 (1.51, 3.02) |  | 2.19 (1.54, 2.96) |
| Missing |  | 82 |  |  |
| **HDL-c(mmol/L)** | 748 |  | 830 |  |
| Median (Q1, Q3) |  | 0.79 (0.44, 1.08) |  | 0.81 (0.45, 1.06) |
| Missing |  | 82 |  |  |
| **WBC(*10^9^/L)** | 830 |  | 830 |  |
| Median (Q1, Q3) |  | 5.09 (3.67, 7.14) |  | 5.09 (3.67, 7.14) |
| Missing |  | 0 |  |  |
| **Hb(g/L)** | 830 |  | 830 |  |
| Median (Q1, Q3) |  | 110 (88, 126) |  | 110 (88, 126) |
| Missing |  | 0 |  |  |
| **PLT(*10^12^/L)** | 829 |  | 830 |  |
| Median (Q1, Q3) |  | 103 (67, 151) |  | 103 (67, 151) |
| Missing |  | 1 |  |  |
| **Hct(%)** | 828 |  | 830 |  |
| Median (Q1, Q3) |  | 32 (27, 37) |  | 32 (27, 37) |
| Missing |  | 2 |  |  |
| **PTA(%)** | 823 |  | 830 |  |
| Median (Q1, Q3) |  | 71 (53, 87) |  | 71 (53, 87) |
| Missing |  | 7 |  |  |
| **PT(s)** | 824 |  | 830 |  |
| Median (Q1, Q3) |  | 15.3 (13.9, 18.1) |  | 15.3 (13.9, 18.1) |
| Missing |  | 6 |  |  |
| **APTT(s)** | 824 |  | 830 |  |
| Median (Q1, Q3) |  | 39 (35, 44) |  | 39 (35, 44) |
| Missing |  | 6 |  |  |
| **TT(s)** | 824 |  | 830 |  |
| Median (Q1, Q3) |  | 18.75 (17.60, 20.30) |  | 18.70 (17.60, 20.30) |
| Missing |  | 6 |  |  |
| **FIB(g/L)** | 824 |  | 830 |  |
| Median (Q1, Q3) |  | 2.44 (1.79, 3.29) |  | 2.46 (1.80, 3.29) |
| Missing |  | 6 |  |  |
| **Antibiotics** | 830 | 131 (16%) | 830 | 131 (16%) |
| Missing |  | 0 |  |  |
| **Insulin dosage** | 830 |  | 830 |  |
| Median (Q1, Q3) |  | 18 (0, 32) |  | 18 (0, 32) |
| Missing |  | 0 |  |  |
| **Duration of diabetes(year)** | 830 |  | 830 |  |
| Median (Q1, Q3) |  | 18 (1, 33) |  | 18 (1, 33) |
| Missing |  | 0 |  |  |
| **BMI(kg/m^2^)** | 774 |  | 830 |  |
| Median (Q1, Q3) |  | 23.4 (21.3, 25.9) |  | 23.7 (21.4, 25.7) |
| Missing |  | 56 |  |  |
| **Hypoglycemia** | 830 |  | 830 |  |
| 0 |  | 712 (86%) |  | 712 (86%) |
| 1 |  | 118 (14%) |  | 118 (14%) |
| Missing |  | 0 |  |  |
| ^1^n (%) | | | | |

**Supplementary Table S2 .**Baseline characteristics in the hypoglycemia and non-hypoglycemia groups

| **Variables** | **all** | **Hypoglycemia** | | ***P*** |
| --- | --- | --- | --- | --- |
|  |  | **No(n= 712)**  ***n(%)/***$\text{M}\text{ (}\text{Q}\text{25, }\text{Q}\text{75)}$ | **Yes(n= 118)**  ***n(%)/***$\text{M}\text{ (}\text{Q}\text{25, }\text{Q}\text{75)}$ |  |
| **Age(year)** | 59 (52, 67) | 59 (52, 67) | 60 (53, 66) | 0.9 |
| **Sex** |  |  |  | 0.6 |
| male | 643 (77%) | 554 (78%) | 89 (75%) |  |
| female | 187 (23%) | 158 (22%) | 29 (25%) |  |
| **Family history of diabetes** |  |  |  | 0.7 |
| No | 773 (93%) | 664 (93%) | 109 (92%) |  |
| Yes | 57 (6.9%) | 48 (6.7%) | 9 (7.6%) |  |
| **Hypertension** |  |  |  | 0.4 |
| No | 547 (66%) | 473 (66%) | 74 (63%) |  |
| Yes | 283 (34%) | 239 (34%) | 44 (37%) |  |
| **Stroke** |  |  |  | 0.7 |
| No | 793 (96%) | 681 (96%) | 112 (95%) |  |
| Yes | 37 (4.5%) | 31 (4.4%) | 6 (5.1%) |  |
| **Cancer** |  |  |  | >0.9 |
| No | 542 (65%) | 465 (65%) | 77 (65%) |  |
| Yes | 288 (35%) | 247 (35%) | 41 (35%) |  |
| **Heart failure** |  |  |  | 0.056 |
| No | 789 (95%) | 681 (96%) | 108 (92%) |  |
| Yes | 41 (4.9%) | 31 (4.4%) | 10 (8.5%) |  |
| **Liver cancer** |  |  |  | 0.5 |
| No | 600 (72%) | 512 (72%) | 88 (75%) |  |
| Yes | 230 (28%) | 200 (28%) | 30 (25%) |  |
| **Hydrothorax** |  |  |  | 0.8 |
| No | 711 (86%) | 611 (86%) | 100 (85%) |  |
| Yes | 119 (14%) | 101 (14%) | 18 (15%) |  |
| **Ascites** |  |  |  | 0.4 |
| No | 379 (46%) | 329 (46%) | 50 (42%) |  |
| Yes | 451 (54%) | 383 (54%) | 68 (58%) |  |
| **Diabetic kidney disease** |  |  |  | <0.001 |
| No | 749 (90%) | 653 (92%) | 96 (81%) |  |
| Yes | 81 (9.8%) | 59 (8.3%) | 22 (19%) |  |
| **Diabetic peripheral neuropathy** |  |  |  | <0.001 |
| No | 763 (92%) | 664 (93%) | 99 (84%) |  |
| Yes | 67 (8.1%) | 48 (6.7%) | 19 (16%) |  |
| **Diabetic peripheral vasculopathy** |  |  |  | 0.007 |
| No | 741 (89%) | 644 (90%) | 97 (82%) |  |
| Yes | 89 (11%) | 68 (9.6%) | 21 (18%) |  |
| **Sulfonylurea** |  |  |  | 0.2 |
| No | 775 (93%) | 668 (94%) | 107 (91%) |  |
| Yes | 55 (6.6%) | 44 (6.2%) | 11 (9.3%) |  |
| **Metformin** |  |  |  | 0.2 |
| No | 706 (85%) | 601 (84%) | 105 (89%) |  |
| Yes | 124 (15%) | 111 (16%) | 13 (11%) |  |
| **Glycosidase inhibitors** |  |  |  | 0.3 |
| No | 714 (86%) | 616 (87%) | 98 (83%) |  |
| Yes | 116 (14%) | 96 (13%) | 20 (17%) |  |
| **SGLT-2** |  |  |  | 0.9 |
| No | 778 (94%) | 667 (94%) | 111 (94%) |  |
| Yes | 52 (6.3%) | 45 (6.3%) | 7 (5.9%) |  |
| **DPP-4** |  |  |  | 0.6 |
| No | 703 (85%) | 601 (84%) | 102 (86%) |  |
| Yes | 127 (15%) | 111 (16%) | 16 (14%) |  |
| **Beta blockers** |  |  |  | 0.12 |
| No | 689 (83%) | 597 (84%) | 92 (78%) |  |
| Yes | 141 (17%) | 115 (16%) | 26 (22%) |  |
| **Diuretic** |  |  |  | 0.2 |
| No | 374 (45%) | 328 (46%) | 46 (39%) |  |
| Yes | 456 (55%) | 384 (54%) | 72 (61%) |  |
| **Hormones** |  |  |  | 0.3 |
| No | 785 (95%) | 676 (95%) | 109 (92%) |  |
| Yes | 45 (5.4%) | 36 (5.1%) | 9 (7.6%) |  |
| **ALT(U/L)** | 33 (22, 65) | 33 (22, 66) | 35 (21, 56) | 0.7 |
| **AST(U/L)** | 47 (31, 88) | 47 (31, 85) | 46 (34, 95) | 0.6 |
| **ALP(U/L)** | 121 (89, 177) | 119 (88, 171) | 133 (99, 195) | 0.007 |
| **rGT(U/L)** | 85 (41, 173) | 84 (39, 173) | 89 (47, 165) | 0.5 |
| **TP(g/L)** | 67 (61, 73) | 67 (61, 74) | 66 (59, 72) | 0.11 |
| **ALB(g/L)** | 32.5 (28.6, 37.1) | 32.7 (28.9, 37.1) | 31.9 (27.5, 35.4) | 0.076 |
| **GLB(g/L)** | 34 (28, 39) | 34 (28, 39) | 33 (28, 40) | 0.7 |
| **PA(mg/L)** | 86 (52, 131) | 86 (54, 132) | 70 (41, 127) | 0.027 |
| **Cr(μmol/L)** | 68 (54, 92) | 67 (54, 90) | 75 (57, 110) | 0.006 |
| **TB(μmol/L)** | 23 (12, 59) | 23 (13, 58) | 22 (10, 72) | 0.6 |
| **DB(μmol/L)** | 9 (4, 28) | 9 (4, 28) | 8 (3, 33) | 0.5 |
| **IB(μmol/L)** | 13 (7, 27) | 13 (7, 27) | 12 (6, 30) | 0.5 |
| **TC(mmol/L)** | 3.95 (3.00, 4.95) | 3.96 (3.02, 4.96) | 3.95 (2.85, 4.90) | 0.6 |
| **TG(mmol/L)** | 1.13 (0.84, 1.63) | 1.15 (0.86, 1.65) | 1.02 (0.77, 1.36) | 0.006 |
| **LDL-c(mmol/L)** | 2.15 (1.51, 3.02) | 2.15 (1.51, 3.03) | 2.10 (1.50, 2.98) | 0.5 |
| **HDL-c(mmol/L)** | 0.79 (0.44, 1.08) | 0.78 (0.45, 1.06) | 0.88 (0.36, 1.23) | 0.3 |
| **WBC(*10^9^/L)** | 5.09 (3.67, 7.14) | 5.06 (3.64, 7.09) | 5.40 (3.87, 7.60) | 0.4 |
| **Hb(g/L)** | 110 (88, 126) | 110 (88, 127) | 108 (88, 123) | 0.3 |
| **PLT(*10^12^/L)** | 103 (67, 151) | 102 (67, 149) | 109 (68, 166) | 0.3 |
| **Hct(%)** | 32 (27, 37) | 32 (27, 37) | 32 (27, 36) | 0.4 |
| **PTA(%)** | 71 (53, 87) | 70 (52, 86) | 74 (54, 90) | 0.2 |
| **PT(s)** | 15.3 (13.9, 18.1) | 15.4 (14.0, 18.2) | 14.9 (13.5, 17.3) | 0.086 |
| **APTT(s)** | 39 (35, 44) | 39 (35, 44) | 38 (33, 44) | 0.2 |
| **TT(s)** | 18.75 (17.60, 20.30) | 18.80 (17.70, 20.30) | 18.50 (17.50, 20.10) | 0.2 |
| **FIB(g/L)** | 2.44 (1.79, 3.29) | 2.39 (1.77, 3.27) | 2.74 (1.95, 3.63) | 0.068 |
| **Antibiotics within 48h** |  |  |  | 0.022 |
| No | 699(84%) | 608(85%) | 91(77%) |  |
| Yes | 131 (16%) | 104 (15%) | 27 (23%) |  |
| **Insulin dosage** | 18 (0, 32) | 16 (0, 32) | 30 (17, 43) | <0.001 |
| **Duration of diabetes(year)** | 18 (1, 33) | 18 (1, 30) | 21 (14, 42) | <0.001 |
| **BMI(kg/m^2^)** | 23.4 (21.3, 25.9) | 23.8 (21.4, 26.0) | 22.3 (20.5, 24.2) | <0.001 |

**Supplementary Table S3 .**Multivariable logistic regression analysis of risk factors associated with hypoglycemia

| **Variable** | **OR** | **95% CI** | **95% CI** | **P Value** |
| --- | --- | --- | --- | --- |
| Creatinine | 1.00 | 1.00, 1.00 | 1.00, 1.00 | **0.027** |
| Indirect bilirubin | 1.00 | 1.00, 1.01 | 1.00, 1.01 | 0.289 |
| Triglycerides | 0.78 | 0.56, 1.10 | 0.56, 1.10 | 0.163 |
| Antibiotics within 48h | 1.50 | 0.80, 2.82 | 0.80, 2.82 | 0.204 |
| Insulin dosage | 1.02 | 1.01, 1.03 | 1.01, 1.03 | **0.002** |
| Duration of Diabetes | 1.01 | 1.00, 1.03 | 1.00, 1.03 | 0.084 |
| BMI | 0.87 | 0.81, 0.94 | 0.81, 0.94 | **<0.001** |

**Supplementary Figure S1.** Density plot comparisons of variables with missing values before and after imputation.


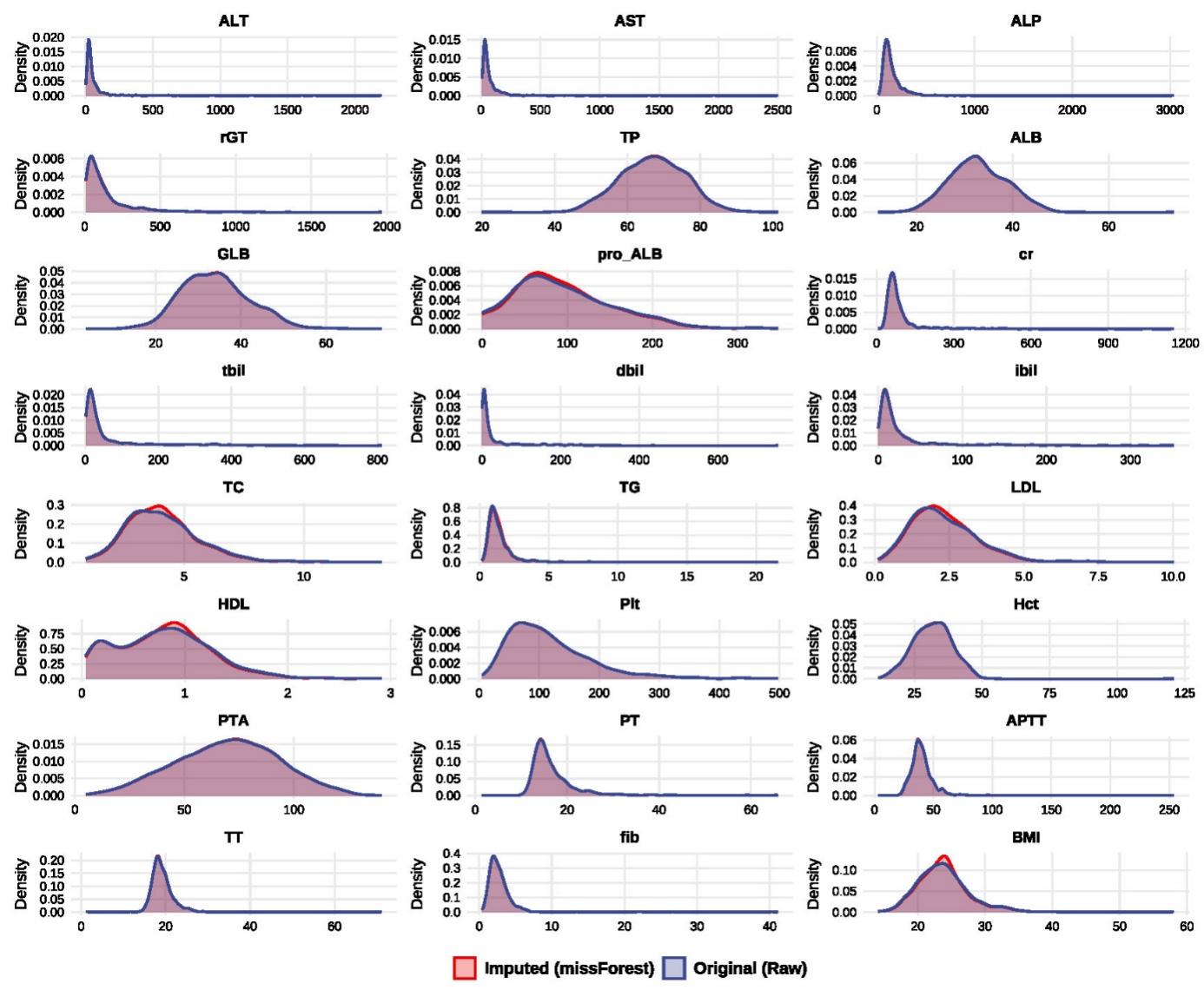

Supplement: Supplementary file 1 — Supporting Information Table S1. Sensitivity analysis comparing the descriptive statistics (median and IQR) of continuous variables before and after random forest imputation. Table S2. Baseline characteristics in the hypoglycemia and nonhypoglycemia groups. Table S3. Multivariable logistic regression analysis of risk factors associated with hypoglycemia. Figure S1. Density plot comparisons of variables with missing values before and after imputation. [file JDR-2026-6812038-s001.docx]
